# The Role of Microglia in Modulating Neuroinflammation after Spinal Cord Injury

**DOI:** 10.3390/ijms22189706

**Published:** 2021-09-08

**Authors:** Sydney Brockie, James Hong, Michael G. Fehlings

**Affiliations:** 1 Division of Genetics and Development, Krembil Research Institute, University Health Network, Toronto, ON M5T 2S8, Canada; s.brockie@mail.utoronto.ca (S.B.); jms.hong@mail.utoronto.ca (J.H.); 2 Institute of Medical Science, University of Toronto, Toronto, ON M5S 1A8, Canada; 3 Spinal Program, Toronto Western Hospital, University Health Network, Toronto, ON M5T 2S8, Canada

**Keywords:** spinal cord injury, microglia, neuroinflammation

## Abstract

The pathobiology of traumatic and nontraumatic spinal cord injury (SCI), including degenerative myelopathy, is influenced by neuroinflammation. The neuroinflammatory response is initiated by a multitude of injury signals emanating from necrotic and apoptotic cells at the lesion site, recruiting local and infiltrating immune cells that modulate inflammatory cascades to aid in the protection of the lesion site and encourage regenerative processes. While peripheral immune cells are involved, microglia, the resident immune cells of the central nervous system (CNS), are known to play a central role in modulating this response. Microglia are armed with numerous cell surface receptors that interact with neurons, astrocytes, infiltrating monocytes, and endothelial cells to facilitate a dynamic, multi-faceted injury response. While their origin and essential nature are understood, their mechanisms of action and spatial and temporal profiles warrant extensive additional research. In this review, we describe the role of microglia and the cellular network in SCI, discuss tools for their investigation, outline their spatiotemporal profile, and propose translationally-relevant therapeutic targets to modulate neuroinflammation in the setting of SCI.

## 1. Introduction

Injury to the spinal cord can be caused by acute trauma or progressive compression from myelopathic mechanisms [1,2]. Degenerative cervical myelopathy (DCM) is the most common form of nontraumatic spinal cord injury (SCI) and occurs with aging, caused by spinal stenosis, spondylosis, and arthritic changes that narrow the spinal canal and impinge on the cord, causing neurologic damage and chronic inflammation [2,3]. Conversely, traumatic SCI may occur spontaneously to any individual, causing instantaneous loss of function or paralysis [1]. In the setting of traumatic SCI, primary injury describes the immediate damage caused by traumatic insult, including spinal contusion or compression, bone fracture, hemorrhage, and membrane disruption. The lesion site becomes littered with necrotic cells and debris, as well as blood and cerebrospinal fluid caused by disruption of the blood spinal cord barrier. Early surgical decompression is the most effective treatment for both forms of SCI to date [1,4], but recovery is limited by the inflammatory response that results from the restoration of blood flow across damaged tissue, causing extensive secondary damage that exacerbates the initial injury [5]. Both phases of inflammation are characterized by well-described neuroinflammatory responses whereby resident microglia and neutrophils respond first, followed by infiltrating monocytes. Together, these phagocytic cells initiate widespread inflammatory cascades through immune cell recruitment and cellular signaling. This review aims to discuss the newest findings regarding the role of microglia in SCI.

## 2. Developmental Origins of Microglia and Advances in Genetic Tools

**Figure 1:**
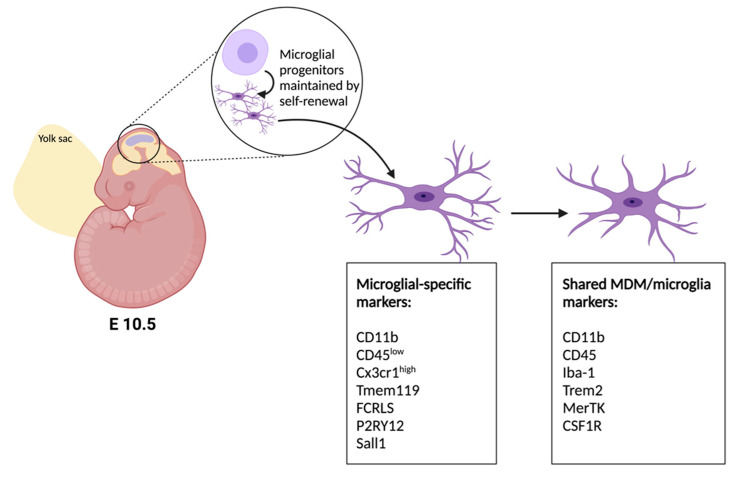
Developmental origins and phenotypic markers of microglia. Neuroepithelial progenitor cells give rise to microglia in mice between embryonic days 10–11. Microglial-specific markers and nonspecific markers of microglia and macrophages [18].

Macrophages and microglia have been assimilated in the past based on shared markers, but their loci of origin and differential gene expression profiles indicate their independence [6] (Figure 1). Microglia are born from the embryonic yolk sac neuroepithelium as progenitor cells, where they develop into resident immune cells of the nervous system by embryonic day 10.5 in mice [7,8] and are maintained throughout life by self-renewal [9], while monocyte-derived macrophages (MDM) develop from blood-borne leukocytes. Moreover, while both cell types express CD11b and CD45 in their activated states, the expression profiles have been distinguished by their relative expression levels (MDM: CD11b + CD45^high^; Microglia: CD11b + CD45^low^) [10]. In the setting of SCI, microglia are known to be activated and upregulate CD45, thereby interfering with microglia-specific targeting [11]. Recently, studies have shown several markers to be microglia-specific, these include Tmem119 [12], Fcrls [13], P2RY12 [14] and Sall1 [15]. Of these, the *Tmem119*, *P2ry12* and *Sall1* genes have inducible *Cre* lines which have enabled further study of microglia-specific roles in central nervous system injury (Table 1). Despite their utility in homeostatic differentiation, not all of these markers have been shown to be stable after injury. For instance, P2RY12 has been shown to be downregulated following SCI [16] and multiple sclerosis [17]–illustrating the gap in current knowledge which necessitates additional research tools and sequencing data to address.

## 3. Heterogeneity of Microglia in the Spinal Cord

In the quiescent state, microglia are dynamic surveyors of the central nervous system (CNS) environment that can be activated to a proinflammatory state involving cytokine signaling, debris phagocytosis, antigen presentation, and immune cell recruitment. Microglia first respond to spinal cord injury within seconds to minutes by extending processes toward the lesion site [22]. These responses display dynamic spatial regulation across grey and white matter regions of the spinal cord. Indeed, post-mortem samples from human cervical SCI patients have demonstrated unequal atrophy between grey and white matter tissue rostro-caudally across the injury site. While grey matter experienced a 30% loss of tissue, white matter showed only a 16.9% loss [23].

Differences in grey and white matter pathology following SCI have been described in terms of hemorrhagic and non-hemorrhagic patterns, where hemorrhaging, defined by blood spinal cord barrier breakdown and vascular leakage, occurs focally in the grey matter and the effects of this bleeding permeates the white matter, yielding secondary effects and region-specific microglial responses [24,25]. Blood spinal cord barrier disruption increases extracellular glutamine and iron levels, consequently contributing to axonal damage in nearby white matter. These differences correlate with differences in microglial profiles in both traumatic and degenerative spinal cord and neural injuries. Further, functional differences in these resident immune cells may in part contribute to the differences observed in biopathology.

In post-mortem tissue samples from human multiple sclerosis lesion sites, white and grey matter microglia (WMM, GMM) were found to have distinct transcriptional profiles across disease progression and the spatial axis [26]. WMM displayed transcriptional upregulation of genes involved in chemotaxis and inflammation, including CXCR4, ACKR1, GPNMB, and NUPR1, followed by lipid metabolism and storage genes, EEPD1, LPL, and PPARG, suggestive of a role for WMM in demyelination and induction of anti-inflammatory regulation [26]. Conversely, grey matter microglia (GMM) displayed transcriptional upregulation of genes involved in glycolysis and iron homeostasis. This suggests GMM are involved in metabolizing the excess iron deposition observed in multiple sclerosis [26], which is energetically costly, necessitating increased glycolytic activity [27]. These findings indicate differences in the roles of WMM and GMM in multiple sclerosis that have similar patterns in other neural injury models.

Parallel themes in regional inflammatory responses have been observed in rat thoracic SCI. One week post-injury, differences existed within and between WMM and GMM, where in the white matter, small, activated microglia were present in the corticospinal tract, along with few macrophages [28]. WMM in the ascending dorsal columns remained ramified and of normal size while in the grey matter, microglia became hyper-ramified and extended branching processes toward the lesion [28]. This response was emphasized in the ventral region, with a significant increase in soma size and cell density and no macrophage presence observed throughout the grey matter [28]. These findings illustrate regional specificity between white and grey matter and within each region itself. More broadly, microglia responses were found to be more profound in white matter than grey. Immunoreactivity was heightened in white matter, indicated by extensive process retraction in activated microglia and recruitment of macrophages [28]. Indeed, in degenerative white matter spinal cord pathologies such as ischemic dementia and multiple sclerosis, WMM are highly activated and proliferative in the early acute phase of disease [29]. This is emphasized at the sites of demyelination, which exist not only in multiple sclerosis, but in traumatic and progressive SCI [30]. In later phases of disease, WMM transition to a reparative role, releasing cytokines such as CXCL10, TGFΒ1, PDGFA, and PDGFB to recruit oligodendrocyte precursor cells [29]. Additional signals such as TGFα, IGF1, and Activin A, which contribute to remyelination, are also secreted by microglia [31,32,33]. These spatiotemporal changes in the transcriptome and secretome of microglia in response to injury can also be observed at rest and in development.

Microglia display region-specific differences and temporal patterns of expression in the contexts of immune response as well as resting states and development. Microglia originate from the embryonic yolk sac where they develop from myeloid precursor cells and are sustained thereafter by self-renewal [34]. Though often conflated for their similar function, this developmental origin is distinct from blood-borne macrophages which develop from monocyte progenitor cells and are not resident to the CNS [34]. Microglia from the cortex (grey matter), cerebellum, and corpus callosum (white matter) have distinct RNA profiles across murine development [35]. At postnatal day 7 (P7), microglia from the corpus callosum displayed an upregulation of phagocytic and chemotactic genes including *Gsn*, *Cd36*, *Abca1*, *Grk6*, and *Scarb1*. At P7, microglia in the corpus callosum also upregulated priming genes SPP1, CLEC7A, CD206 in Iba-1-positive, actively proliferating microglia, and those in the cerebellum displayed similar upregulation of SPP1. These profiles were lost by P10 in both regions and microglia from the corpus callosum transitioned to apoptotic and necrotic gene profiles, while cortical microglia, by contrast, were found to be steadily growing with little activation by P7 and P10, and maintained this quiescent phenotype thereafter. This suggests general differences in basic cell progression, where grey matter microglia are slow-building and quiescent, while white matter microglia are highly proliferative and activated early on, followed by more exaggerated, persistent pruning periods. Similarly, *Trem2*, which encodes the triggering receptor expressed on myeloid cells 2 molecule that is involved in modulating the microglial immune response, has been found to display differential expression across brain regions in postnatal development [36]. Specifically, in the subventricular zone and neuroepithelium, expression peaked between P5–7, while in the corpus callosum, expression increased from P3–7, and in the fimbria, expression peaked at P3, after which points, expression sharply declined and was undetectable after P10 across all regions [36]. Similarity in region-specific expression patterns across development and neuroinflammation represent innate microglial characteristics that contribute to functional specialization of cell populations.

## 4. Role of Microglia Following SCI

**Figure 2:**
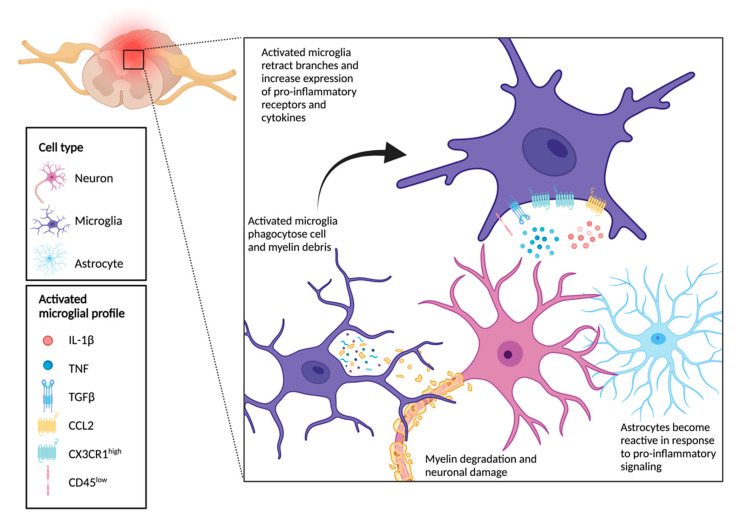
Microglial functional profile in neuroinflammation.

Microglia, the resident immune cells of the CNS, respond first to injury signals in conjunction with auto- and paracrine signaling from astrocytes and neurons by initiating widespread inflammatory cascades [37]. Within minutes, microglia take on a proinflammatory state [38] where they become amoeboid-like phagocytes that clear cell and myelin debris and recruit immune cells via cyto- and chemokine signaling (Figure 2) [39]. Activated and quiescent microglial phenotypes have been described in the past as M1 and M2 inflammatory and alternative polarized states, respectively [40]. Current research suggests this may be a false dichotomy, however, as several canonical markers of each state are highly co-expressed in single cells [41]. These states may instead represent a continuum of microglial phenotypes, paralleled by MDM, which are dynamically regulated by spatial and temporal factors and according to injury severity [42].

In activated states, cytokines released by microglia include IL-1, IL-6, IL-12, IL15, IL-23, interferon-γ (IFN-γ), and tumor necrosis factor (TNF), which elicit inflammatory responses from nearby cells [43]. Additionally, chemokines, expressed as membrane-bound or soluble proteins, include CCL8, CCL15, CCL19, CCL 20, CXCL9, CXCL10, CXCL11, and CXCL13 [43], which act as homing signals to circulating leukocytes, B cells, T cells, natural killer cells, dendritic cells, and macrophages [44]. Further, as laminar flow is reduced in the early inflammatory response, circulating immune cells are allowed greater contact with endothelial lining [45] and are subsequently recruited by these chemoattractants to the injury site. Reactive nitric oxide species, damage-associated molecular patterns, and inflammatory signals then facilitate their extravasation into the adjacent damaged tissue where they aid in pathogen defense and phagocytosis [43].

MDM begin to infiltrate the lesion site within two to three days [46], at which point IL-10-expressing cells aid in the execution of the immune response and phagocytosis of myelin debris and fragmented cell components [47]. By day 14, MDM become the chief modulators of phagocytosis [46], though microglia may prove more efficient at processing this material, indicated by the fact that, at 42 days post-SCI, debris can still be found in MDM, but not microglia [48]. Further, once they’ve arrived at the injury site, MDM attenuate microglial expression of IL-1β, TNF, and IL-6 [49]. Moreover, MDM do not negate microglial function, but rather facilitate its phenotypic shift.

Mammals express approximately 20 different connexins, which are the primary constituent of gap junctions, and 3 pannexins. In both classes of these gap junctions, each channel is formed by two hemichannels. After SCI, both activated microglia and astrocytes have been demonstrated to release excessive glutamate through gap junction hemichannels—ultimately contributing to glutamate excitotoxicity. Studies in contusive preclinical models of SCI using both connexin inhibitors such as INI-0602 [50], Cx43-asODN (connexin-43 anti-sense oligonucleotide) [51], and Peptide 5 (connexin-43 mimetic peptide) [52] have observed improved neuroinflammation, electrophysiology, and functional recovery. To date, there have not been any studies conducted that investigate the role of pannexins in SCI pathophysiology.

Hypoxia and ischemia are both critical components of secondary injury in SCI; studies [24,53,54,55] have demonstrated that hypoxia and ischemia-mediated transient vascular leakage is associated with the activation and accumulation of microglia around vessels. Pharmacological depletion of microglia using PLX5622, a CSF-1 inhibitor, had no effect in normoxic conditions, but resulted in profound vascular leakage due to a loss of tight junction proteins in the endothelium. Further, using a blocker of fibrinogen and microglial integrin receptor Mac-1, microglia-mediated vascular repair/maintenance was hindered. Another study looked to bias microglial polarization towards the M2 state using tetramethylpyrazine and found that TMP-treated M2 microglia through activation of STAT3/SOCS3 and inhibition of NK-κB promote blood-spinal cord barrier integrity in EAE mice [56]. Taken together, these studies demonstrate the importance of microglia in the maintenance and repair of the blood-spinal cord barrier.

## 5. Chronic Inflammation Following SCI

Vasoconstriction caused by cytokine signaling following SCI imposes increased stress on vessels, compromising their structural integrity and contributing to prolonged vascular leakage over time [57]. Prolonged cytokine activity contributes to the development of chronic inflammatory conditions including metabolic, autoimmune, and neurologic disorders [57]. Elevated cytokine levels can disrupt the hypothalamic-pituitary-adrenal axis (HPA) by increasing efferent signaling and causing hyperactive hormone production [58]. Similarly, severance of afferent nerve fibers may also cause HPA hyperactivity, whereby feedback signaling is inhibited, resulting in unabated adrenal hormone production [58]. Given the close relationship between the endocrine and innate immune systems, disruptions in neuronal signaling that alter hormone production either acutely or chronically have potent effects on recovery and long-term outcomes. Cortisol and norepinephrine represent two of several key endocrine effectors that, when disrupted, may cause suppression or hyperactivation of the innate immune system and hinder long-term recovery.

Neuronal activity is acutely impacted by SCI at or above the T3 level, as this disrupts autonomic lymphoid innervation at the spleen, downregulating the production and release of anti-inflammatory catecholamines such as norepinephrine [59]. Conversely, injury at T9 appears to leave splenic sympathetic ganglia intact, preserving B cell activation, production of autoantibodies, and auto-activation of T cells [60]. Natural killer cell and lymphocyte activity, however, are shown to be affected independent of the spinal lesion site and subsequent immune signaling and neural recovery are impaired [61,62].

## 6. Crosstalk between Microglia, Neurons, and Astrocytes Drives Functional Outcomes Following SCI

Microglia and macrophages dynamically modulate injury response through a variety of mechanisms including their participation in corralling and wound compaction via Plexin-B2 signaling [63]. Increased expression of Plexin-B2 in activated microglia and macrophages early in injury progression limits their diffusion from the lesion site, thereby focusing their effect and attenuating widespread tissue damage [63]. Additionally, in a lipopolysaccharide-induced model of inflammation, microglia-depleted mice showed reduced astrocyte reactivity than wildtype controls [64] via Il-1α, TNF, and C1q signaling [65]. It has also been suggested that astrocytic withholding of cholesterol can act as an inhibitory signal to microglia [65], thus establishing feedback control. Following SCI, astrocytes can be both beneficial and harmful, forming a protective barrier around the lesion site, but also exerting cytotoxic effects on neurons and mature oligodendrocytes in their reactive state. Under normal conditions, GFAP-expressing astrocytes in the spinal cord predominantly localize to white matter. Following SCI, astrocytes in the outer white matter hypertrophy and infiltrate the grey matter to form a thick glial scar around the lesion site, protecting neuronal tracts from toxicity following blood barrier breakdown. Indeed, animal models of SCI where astroglial scarring is inhibited demonstrate larger lesion cavities and increased neuronal death [66].

On the other hand, excitotoxicity has been found to play a major role in secondary cell death, constituted by a spike in glutamate release from injured astrocytes, microglia and neurons that results in lethal ionic disruption in postsynaptic cells [67,68]. Elevated glutamatergic signaling and glial cell depolarization causes influx of calcium, leading to production of nitric oxide and reactive oxygen species in postsynaptic neurons and oligodendrocytes [53]. Subsequent loss of oligodendrocytes may then contribute to the spontaneous degeneration of spared axons surrounding the lesion site [69,70].

Neuronal preservation largely determines functional recovery after SCI. Microglia-neuronal crosstalk is significantly mediated by fractalkine (FKN) signaling [71]. In the CNS, the FKN receptor, CX3CR1 is predominantly expressed by microglia and monocytes and to a lesser extent, natural killer and dendritic cells [71]. Its ligand, CX3CL1, is expressed primarily by neurons, and can exist in membrane-bound and soluble isoforms and is a major mode of communication between these cell pools [72,73]. The interaction of membrane-bound FKN with its receptor functions to maintain microglial surveillance during quiescence. In injury, CX3CL1 is cleaved from its membrane-bound glucose by ADAM family metalloproteases, where it acts as a ‘find me’ signal that attracts CX3CR1-expressing microglia and monocytes to which it then binds and induces activation binds to microglial receptors in its soluble isoform and induces their activation [74,75]. CX3CR1 has thus been found to be upregulated in injury, resulting in increased microglial/monocyte activation and proinflammatory signaling [76]. Genetic deletion of CX3CR1 has been found to improve functional recovery in mice after cerebral ischemia by reducing microglial activation and thereby attenuating secondary injury [77]. Given the spatial separation of neurons and astrocytes, microglia are a necessary liaison in the crosstalk between glia and the functional network. Moreover, in addition to secreting factors and direct membrane-bound contact, microglia also receive signals from neurons and astrocytes, establishing a dynamic feedback relationship between these cell populations [65]. These networks and the interactions that mediate them are attractive therapeutic targets for inflammation-focused treatment of SCI.

## 7. Therapeutic Targeting of Microglia after SCI

Given the role of microglial as the resident immune cells of the nervous system, many efforts have been made to target their activation so as to attenuate inflammatory injury (Table 2). In ischemic injury as well as traumatic brain and spinal cord injury models, functional recovery has been achieved by inhibiting fractalkine signaling [75,77,78]. Genetic knockout as well as pharmacologic inhibition of this receptor or its ligand in models of neural injury have been found to reduce lesion size and increase axonal sparing by attenuating microglial/macrophage activation, thus promoting functional recovery [71,77,78].

Similarly, colony stimulating factor 1 receptor (CSF1R) has also been used to target microglia and macrophages in CNS injury. Its ligand, CSF1, is a secreted ligand that modulates microglia and macrophage activation and cytotoxicity [79]. While its genetic deletion results in several developmental abnormalities and deficits [80], pharmacologic inhibition of CSF1R has been found to be effective in treating SCI. Inhibition of CSF1R using GW2580 has been found to significantly reduce activation and proliferation of these cells following SCI, attenuating inflammation and apoptosis and resulting in improved fine motor recovery [81]. Similarly, PLX5622 has also been used to successfully inhibit CSF1R in cases of neuropathic pain [82].

In SCI, activated microglia and astrocytes secrete thrombospondins that bind to the alpha2delta (α2δ) subunit of calcium channels and cause excitatory synaptogenesis. Gabapentin, a US Food and Drug Administration approved drug, has been used in the treatment of SCI for its ability to bind to the α2δ subunit to block synaptogenic excitotoxicity [83]. Specifically, it has been used to successfully attenuate spasticity [84], neuropathic pain [85,86], and autonomic dysreflexia [87]. Most recently, Brennan and colleagues used the human-equivalent daily dose of 1.1 gram of gabapentin as a prophylactic treatment for wildtype mice subjected to a thoracic level 3 crush injury and were successful in preventing deleterious synaptic plasticity [87]. By blocking excitatory signaling, gabapentin reduced aberrant neuronal activity, thereby preventing autonomic dysreflexia and immune suppression. This application of gabapentin as well as those studied previously offer approaches to target microglia in the preventative and symptomatic treatment of SCI.

In addition to blocking maladaptive effects of microglial activity, efforts have been made to encourage their therapeutic capacity by biasing microglia toward to a traditionally anti-inflammatory phenotype for subsequent transplant into the injured spinal cord [88]. Microglia were treated with either granulocyte-macrophage CSF or interleukin-4 to generate M1 and M2 microglia, respectively. These microglia were then injected into wildtype mice that had been subjected to SCI at the thoracic 12 and 13 levels. Motor function and hindlimb reflexes were significantly improved in the M2-treated group, with similar benefits recapitulated at the cellular and molecular levels. mRNA and protein analysis illustrated decreased expression of proinflammatory markers CD86, and RNOS-related enzymes and increased expression of insulin-like growth factor (Igf1) in the M2-treated group [88]. These findings highlight novel approaches to optimizing microglial function in SCI and a potential avenue for cell therapy in its treatment.

Microglia are integral components in many cell networks in the CNS, making them an attractive therapeutic target in SCI and neural injury [89]. Their vast cell-to-cell communicability, however, makes them complex nodes in these networks with numerous downstream effects. For this reason, no one treatment approach will be sufficient to abrogate their effect altogether, nor is it obvious that this would be desirable. There thus remains a need for continued research into the complex and dynamic functions of microglia in order to optimize current treatment of SCI and ultimately improve functional recovery.

## 8. Conclusions

In summary, SCI is a complex condition that can be caused by a variety of mechanisms. It involves a primary injury phase, characterized by physical contusion, crush, or compression of the spinal cord and the instantaneous necrotic cell death that follows. Vascular disruption perpetuates this injury by initiating an inflammatory response in the tissue emanating from the initial lesion site. This causes a second phase of injury, characterized by inflammation, apoptotic and necrotic cell death, and Wallerian degeneration. The inflammatory response that ensues is mediated by both resident and infiltrating immune cells.

Microglia are primary regulators of the innate immune response in the CNS, making them attractive therapeutic targets. This cell population is dynamic in its signaling modalities and spatiotemporal specialization. In order to improve functional recovery from SCI, it is necessary to understand the complex nature of these cells so that we may target them effectively to reduce their damaging inflammatory effects while harnessing their regenerative capabilities. Future research should focus on investigating the heterogeneity of microglial populations and developing treatment strategies that employ multi-faceted approaches to target primary and secondary injury substrates.

## Figures and Tables

**Table 1:** Commercially available microglial marker-specific Cre-inducible mouse strains.

Marker	Strain	Original Publication
Tmem119	C57BL/6-Tmem119^em1(cre/ERT2)Gfng^/J	[19]
P2ry12	B6(129S6)-P2ry12em1(icre/ERT2)Tda/J	[20]
Sall1	Sall1tm5(cre/ERT2)Ryn	[21]

**Table 2:** Summary of discussed therapeutic interventions for microglial-mediated neural injury. M2: anti-inflammatory microglia.

Target	Therapeutic Intervention	Original Publications
CX3CR1/CX3CL1	Genetic deletion	[71,77]
CSF1R	Pharmacologic inhibition (GW2580, PLX5622)	[80,81]
Ca^2+^ channel α2δ subunit	Pharmacologic inhibition (Gabapentin)	[83,84,85,86]
M2 microglia	IL-4 biasing of M2 cells for transplant	[87]
